# Programmed death 1 inhibitor combined with radiotherapy decreases epidermal growth factor receptor expression in breast cancer

**DOI:** 10.1186/s41065-025-00472-x

**Published:** 2025-08-16

**Authors:** You Wu

**Affiliations:** School of Medicine, Nanjing University of Chinese Medicine, Nanjing, 210023 Jiangsu Province China

**Keywords:** PD-1 inhibitors, Radiotherapy, P53, EGFR, BC

## Abstract

**Objective:**

To investigate the effect of programmed death 1 (PD-1) inhibitors combined with radiotherapy on the expression of P53 and epidermal growth factor receptor (EGFR) in breast cancer (BC). The impact of radiation treatment on BC patients’ prognosis was examined.

**Methods:**

The data of BC patients admitted to Nanjing University of Chinese Medicine from April 2022 to April 2023 were retrospectively analyzed. The clinical data of the patients were extracted, and 104 patients were randomly enrolled. The survival time and complications of patients were followed up. The expression levels of P53 and EGFR in tumor tissues and prognosis of patients treated with PD-1 inhibitor combined with radiotherapy were analyzed.

**Result:**

The expression level of EGFR in the joint group (JG) was visibly lower as against the control group (CG); The median progression-free survival (PFS) of the JG was visibly longer as against the CG (all *P <* 0.05). The optimal cut-off values of P53 positive rate and EGFR level before combined treatment were 10% and 96.21ng/mL. In addition, the median PFS of individuals with low EGFR in the JG was 7.3, which was visibly higher as against individuals with high EGFR. The level of EGFR before PD-1 inhibitor treatment was an independent cause of risk affecting the prognosis of patients.

**Conclusion:**

PD-1 inhibitor plus radiotherapy can effectively inhibit the expression of EGFR in tumor tissues of BC patients and visibly improve the prognosis.

**Clinical trial number:**

Not applicable.

## Background

BC is one of the most common female malignancies worldwide, causing a visible health burden among women. According to the WHO, the incidence of BC has been on the rise in most countries since 2018, and among patients aged ≥ 50 years, Japan, Slovakia, and China have the most prominent increase in incidence reports [1]. The risk factors of BC include lifestyle, genetic and environmental factors. Some studies have also suggested that alcohol can contribute to the risk of BC [2]. In the treatment of BC, surgery, radiotherapy and chemotherapy, targeted therapy, and other methods can improve the survival rate of patients. Immunotherapy is a research hotspot of cancer treatment, which can improve the microenvironment of tumor tissue, thereby improving the immune function of patients, to block the development of cancer [3]. Single radiotherapy has certain limitations in the treatment of cancer, and long-term radiotherapy may cause serious adverse reactions and drug resistance. PD-1 inhibitors plus radiotherapy have shown good efficacy in the clinical treatment of BC, but the effect on the expression of P53 and EGFR in BC has not been elucidated [4, 5]. Therefore, the research on the expression of P53 and EGFR and prognosis analysis of BC has important clinical significance and scientific value.

PD-1 inhibitors have attracted much attention in the immunotherapy of cancer. Clinical studies have shown that antibodies targeting the immune checkpoint of the PD-1/PD-L1 pathway have a good effect on the treatment of cancer diseases [6]. PD-1 is an immune checkpoint molecule. Most studies have shown that PD-1/PD-L1 signaling pathway blockade therapy can activate the immune ability of cytotoxic T effector cells to BC cells. PD-1 inhibitors promote the immune function of T cells to tumor cells by inhibiting the interaction between PD-1 and its ligand PD-L1 to achieve the therapeutic effect [7]. At present, there are many PD-1 inhibitors available in clinical practice, including Pembrolizumab and Tislelizumab injection. Pembrolizumab monotherapy has been shown in certain studies to be effective in treating advanced non-small cell lung cancer patients by slowing down the progression of the illness and increasing the patients’ overall survival (OS) and median PFS [8]. Some studies have also found that pembrolizumab and pembrolizumab chemotherapy continue to show more visible survival benefits than traditional chemotherapy in recurrent/metastatic head and neck squamous cell carcinoma [9]. The risk of recurrence or death in patients with stage IIB/C melanoma treated with pembrolizumab for 1 year was visibly reduced [10]. In a meta-analysis of pembrolizumab in the treatment of triple-negative BC (TNBC), it was found that the objective response rate (ORR) of patients with BC treated with pembrolizumab plus chemotherapy was visibly higher as against the traditional placebo group, and the PFS of patients could be enhanced [11]. Some studies have found that the therapeutic sensitivity of pembrolizumab is related to the P53 pathway [12]. P53 is a tumor suppressor gene that is involved in several critical biological processes, including apoptosis, DNA repair, and cell cycle control [13, 14]. Its mutation or dysfunction is common in a variety of tumor diseases such as BC, and is closely related to the development and prognosis of tumors. EGFR participates in the process of cell growth, proliferation, and differentiation. In BC, the abnormal expression of EGFR is related to the invasiveness and prognosis of the tumor, and it is considered an important therapeutic target. Studies have proposed a lack of estrogen (ER) and progesterone receptor (PgR) as well as EGFR in TNBC patients [15]. Pembrolizumab plus chemotherapy has shown a clear benefit in terms of patient survival and is approved as a first-line treatment for EGFR-positive tumors in the United States [16].

In summary, PD-1 inhibitors plus radiotherapy, as an emerging treatment strategy, has potentially important implications for the treatment of BC. P53 and EGFR, as the key molecular markers in the occurrence and development of BC, play a major role in PD-1 inhibitors plus radiotherapy. Its goal was to find out how PD-1 inhibitors plus radiotherapy worked on the expression of P53 and EGFR and prognosis of BC, providing new ideas and clinical guidance for the treatment of BC.

## Methods

### General data

The data of BC patients admitted to Nanjing University of Chinese Medicine from April 2022 to April 2023 were retrospectively collected, and 104 patients were randomly included. According to the treatment method, the patients were divided into control group (CG, *n* = 54) and joint group (JG, *n* = 50). BC patients were diagnosed by pathology (42 Luminal subtype, 31 HER2-positive subtype, and 31 triple-negative subtype). EGFR expression was fully assessed in pre-treatment biopsy samples (with a positive result defined as an immunohistochemical score of ≥ 2+, where the positivity rates were 58.3% for the Luminal subtype, 67.7% for the HER2-positive subtype, and 80.6% for the triple-negative subtype).

Inclusion criteria: patients were diagnosed with BC; no history of PD-1 inhibitor use or radiotherapy treatment; the clinical data of the patients were complete, including imaging examination before and after treatment and follow-up information at 12 months after treatment; patients with heart, liver, and renal insufficiency; patients receiving PD-1 inhibitors plus radiotherapy or radiotherapy alone.

Disqualification: individuals with a history of other tumors; individuals with immunodeficiency diseases; individuals who were lost to follow-up; the clinical data were incomplete.

### Treatment methods

The CG received radiation therapy alone. All patients were first positioned for scanning using the Ingenuity CT scanner (Model: Ingenuity Core 128) manufactured by Philips Medical Systems (Netherlands). Patients were positioned in the standard supine position with both arms raised and fixed, with a slice thickness of 3 mm covering the entire breast and regional lymph nodes. Radiation therapy was delivered using the Clinac iX linear accelerator (Model: Clinac iX 2300) manufactured by Varian Medical Systems (USA). The treatment protocol involved whole-breast irradiation at 50 Gy in 25 fractions (2 Gy per fraction, five times per week), followed by an additional 10–16 Gy in 5–8 fractions to the tumor bed, with the specific dose determined based on the surgical margin status. The treatment employed either three-dimensional conformal radiation therapy (3D-CRT) or intensity-modulated radiation therapy (IMRT) techniques, with strict adherence to normal organ dose constraints (ipsilateral lung V20 < 30%, average cardiac dose < 8 Gy).

The JG received PD-1 inhibitor in combination with radiation therapy. The radiation therapy protocol for JG was identical to that of the CG, also using the Varian Clinac iX linear accelerator (Varian Medical Systems, USA). The PD-1 inhibitor treatment protocol included one of the following agents, selected based on the patient’s condition: pembrolizumab (200 mg every 3 weeks, manufactured by Merck, USA), nivolumab (240 mg every 2 weeks, manufactured by Bristol-Myers Squibb, USA), sintilimab (200 mg every 3 weeks, manufactured by Innovent Biologics, China), or camrelizumab (200 mg every 2 weeks, manufactured by Henlius Biopharma, China). The first dose was administered within 7 days prior to the start of radiation therapy, and regular dosing continued throughout the treatment period until 1 year post-radiation or the occurrence of intolerable toxicity. All patients received 20 mg of diphenhydramine by intramuscular injection 30 min before each dose to prevent allergic reactions, and an additional 5 mg dose of dexamethasone was administered intravenously during the first infusion.

A treatment safety monitoring protocol was implemented throughout the entire treatment process. Both groups of patients underwent weekly toxicity assessments using the *CTCAE v5.0* criteria. Patients in the combination group also had weekly monitoring of complete blood count, liver and kidney function, and thyroid function. Radiation therapy was paused if grade 3 radiation dermatitis or grade 2 radiation pneumonitis occurred, and PD-1 inhibitor treatment was permanently discontinued in the event of ≥ grade 3 immune-related adverse reactions. There was a minimum interval of 24 h between PD-1 inhibitor administration and radiation therapy to ensure safety and efficacy. All treatment equipment underwent regular quality control and calibration by the hospital’s medical engineering department.

### Collection of data

Patient data included gender, age, pathological stage, pathological grade, pathological type, P53and EGFR expression level, and underlying diseases.

In determining the threshold values for P53 and EGFR expression levels, standardized analytical methods were employed. For P53 expression (immunohistochemical detection, clone DO-7), the threshold value was determined through ROC curve analysis, using progression-free survival (PFS) at 12 months as the endpoint. The threshold value corresponding to the maximum Youden index was 10% (AUC = 0.713, sensitivity = 78.63%, specificity = 70.12%). For EGFR levels (ELISA detection, R&D Systems kit), a restricted cubic spline model was used to analyze the nonlinear relationship with treatment response, with the inflection point of the curve at 96.21 ng/mL selected as the threshold value (AUC = 0.697, sensitivity = 63.32%, specificity = 69.21%). All measurements were conducted using primary tumor tissue samples obtained via pre-treatment core needle biopsy and were double-blind reviewed.

For the assessment of adverse reactions, a standardized approach combining biochemical tests and clinical evaluations was employed. Hepatotoxicity was defined as ALT/AST > 3 times the upper limit of normal or total bilirubin > 2 times the upper limit of normal (according to *CTCAE v5.0* criteria); Renal toxicity was assessed based on serum creatinine (increase > 1.5 times baseline) and GFR (calculated using the CKD-EPI formula, decrease > 25%); Gastrointestinal dysfunction was determined according to the *CTCAE v5.0* symptom grading criteria; Hematologic toxicity (including thrombocytopenia, anemia, and leukopenia) was quantitatively assessed through complete blood cell analysis (Sysmex XN-1000 analyzer). All assessments were conducted in a standardized manner before treatment, weekly during treatment, and 4 weeks after treatment, with independent confirmation by two physicians (Kappa > 0.80).

### Evaluation criteria

The Immune Response Evaluation Criteria in Solid Tumors (iRECIST) was employed. Adverse events were evaluated based on the Common Terminology Criteria (CTCAE) Version 5.0 (NCI CTC 5.0).

### Follow-up

Patients were followed up by telephone, Wechat, and electronic medical record for disease progression within 12 months after treatment.

### Statistical methods

Excel and SPSS 27.0 data analysis software were employed. Data presented as mean ± sd ($\:\overline{\text{x}}$±s) were compared by *t* test. Data presented as cases (percentage) [n(%)] were subjected to contrast by chi-square test. The Kaplan-Meier (KM) method was employed for analyzing the PFS of patients. The receiver operating characteristic (ROC) curve was adopted to calculate the optimal cut-off values of P53 and EGFR before PD-1 inhibitor treatment. The significance level was set at *P <* 0.05.

## Results

### General clinical data of the patients

In Table 1, the CG and the JG were well balanced in terms of baseline characteristics. No statistically significant differences were observed between the two groups in key baseline indicators, including age distribution (> 45 years: CG 24.1% vs. JG 24.0%, *P* = 0.87), pathological staging (stage I/II: CG 59.3% vs. JG 60.0%, *P* = 1.36), and histological grading (grade III: CG 50.0% vs. JG 50.0%, *P* = 1.67) (*P* > 0.05). Additionally, the distribution of major pathological subtypes, including ductal carcinoma in situ (DCIS), invasive ductal carcinoma (IDC), and invasive lobular carcinoma (ILC), was comparable between the two groups. The incidence of comorbidities (CG 13.0% vs. JG 12.0%) and metastatic status (CG 72.2% vs. JG 74.0%) also showed no significant differences.

**Table 1:** General data of the patients

General information	CG (*n* = 54)	JG (*n* = 50)	χ^2^	*P*
Age			0.15	0.87
> 45 (years old)	13	12		
≤ 45 (years old)	41	38		
Pathological staging			0.01	1.36
I / II	32	30		
III / IV	22	20		
Pathological grading			0.02	1.67
I / II	27	25		
III	27	25		
Pathological type			0.46	7.84
DCIS	17	18		
IDC	26	24		
ILC	8	6		
Other	3	2		
Basic diseases			0.05	2.93
Yes	7	6		
No	47	44		
Transfer or not			0.04	2.89
Yes	39	37		
No	15	13		

Note: DCIS: ductal carcinoma in situ; IDC: invasive ductal carcinoma; ILC: invasive lobular carcinoma

### Expression of P53 and EGFR in BC patients

**Fig. 1:**
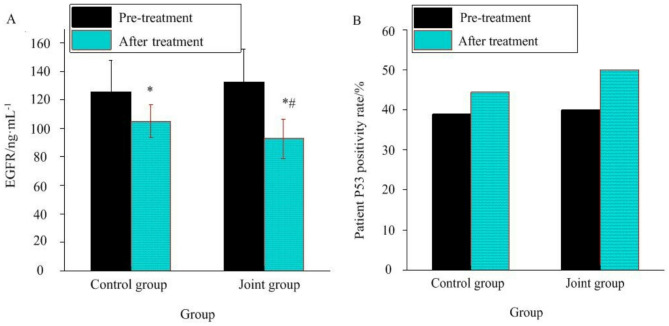
Expression of P53 and EGFR in BC patients before and after treatment. (**A**: EGFR; **B**: P53). Note: *As against that before treatment, #As against the CG, *P <* 0.05

The expression changes of P53 and EGFR in BC patients before and after treatment were analyzed. There was no significant variation in P53 and EGFR between the two groups before treatment (*P >* 0.05). Through treatment, EGFR in the JG was visibly lower as against the CG (*P <* 0.05). However, there was no significant variation in the positive rate of P53 in BC tissues before and after treatment (*P >* 0.05) (Fig. 1).

### Optimal levels of P53 and EGFR before PD-1 inhibitor treatment

**Fig. 2:**
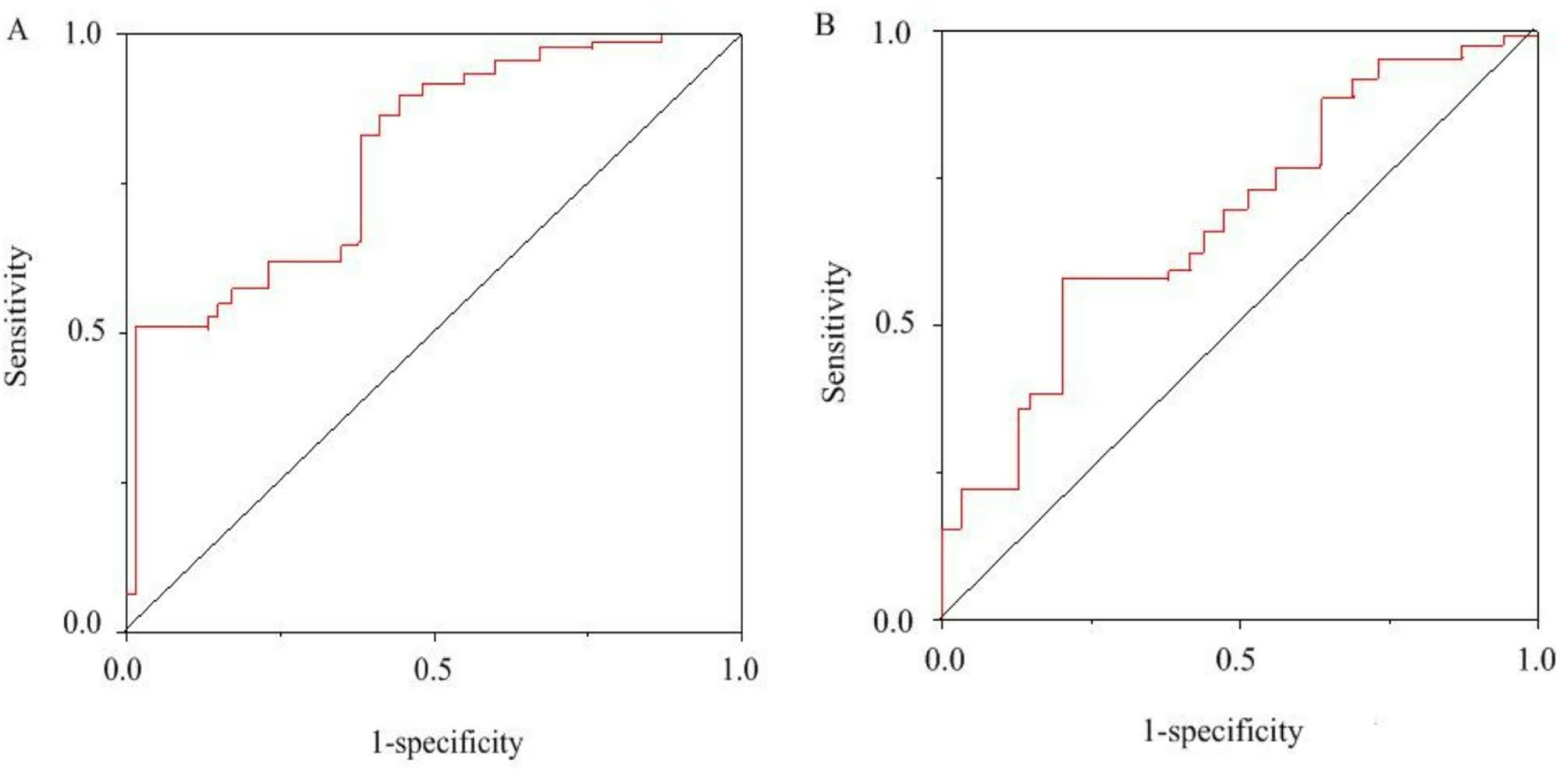
ROC curve analysis of optimal levels of P53 and EGFR prior to PD-1 inhibitor treatment. (**A** is P53; **B** is EGFR)

Figure 2 illustrates the ROC curve analysis of the optimal levels of P53 and EGFR prior to PD-1 inhibitor treatment. The area under the curve (AUC) of P53 was 0.713, the maximum Youden index was 0.362, the sensitivity and specificity were 78.63% and 70.12%, respectively. The AUC of EGFR was 0.697, the maximum Youden index was 0.372, and the sensitivity and specificity were 63.32% and 69.21%, respectively. According to the intercept value, P53 < 10% (low positive rate), P53 ≥ 10% (high positive rate), EGFR < 96.21ng/mL (low level), EGFR ≥ 96.21ng/mL (high level).

### Analysis of PFS after PD-1 inhibitor plus radiotherapy

**Fig. 3:**
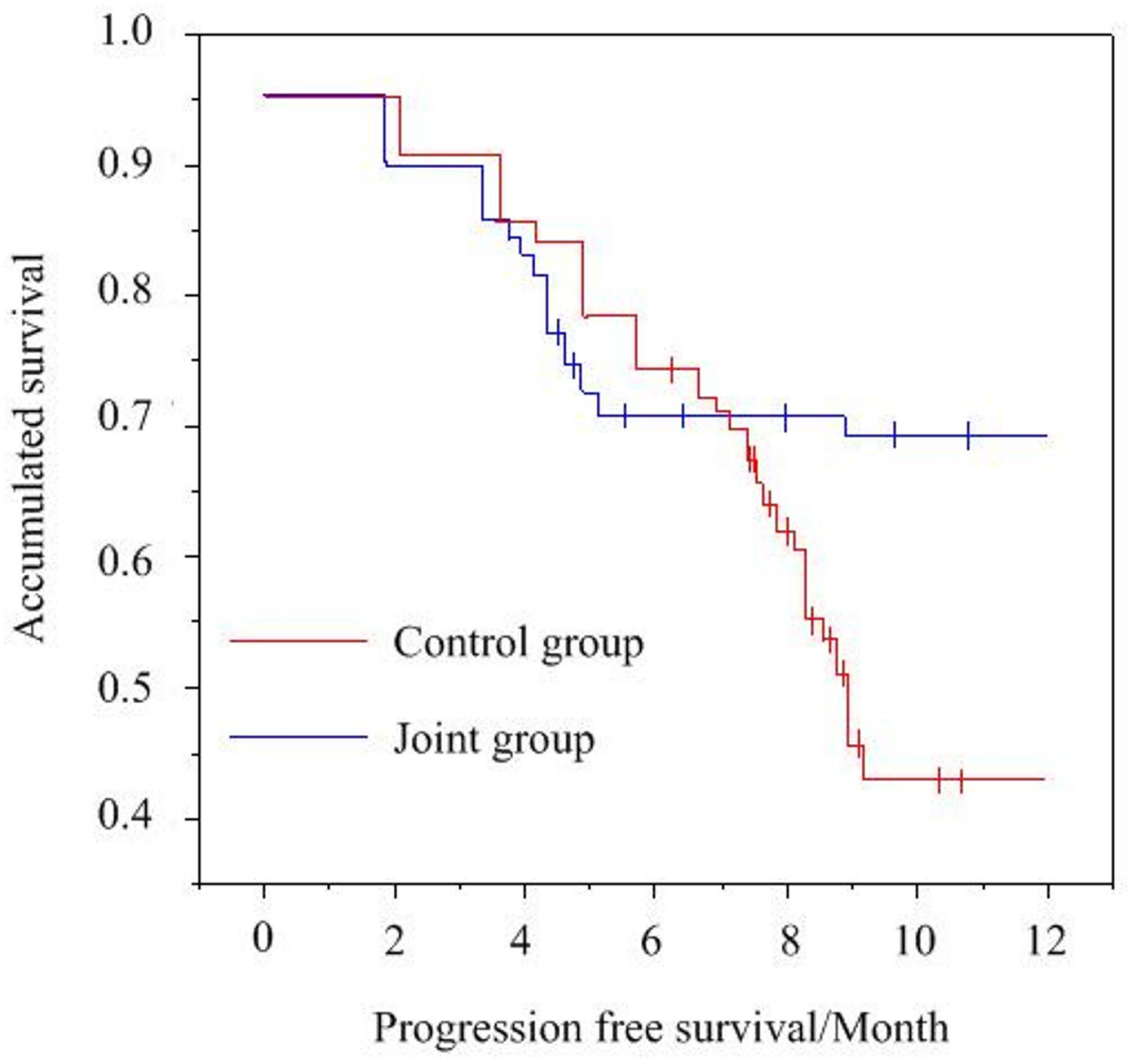
Survival curve of PFS

The survival curve of PFS by KM method is illustrated in Fig. 3. As against the CG [5.4 months (4.2–7.1 months)], the median PFS of the JG [8.2 months (6.3–8.7 months)] was visibly prolonged (*P <* 0.05).

### Effect of PD-1 inhibitor plus radiotherapy on patients after treatment

**Fig. 4:**
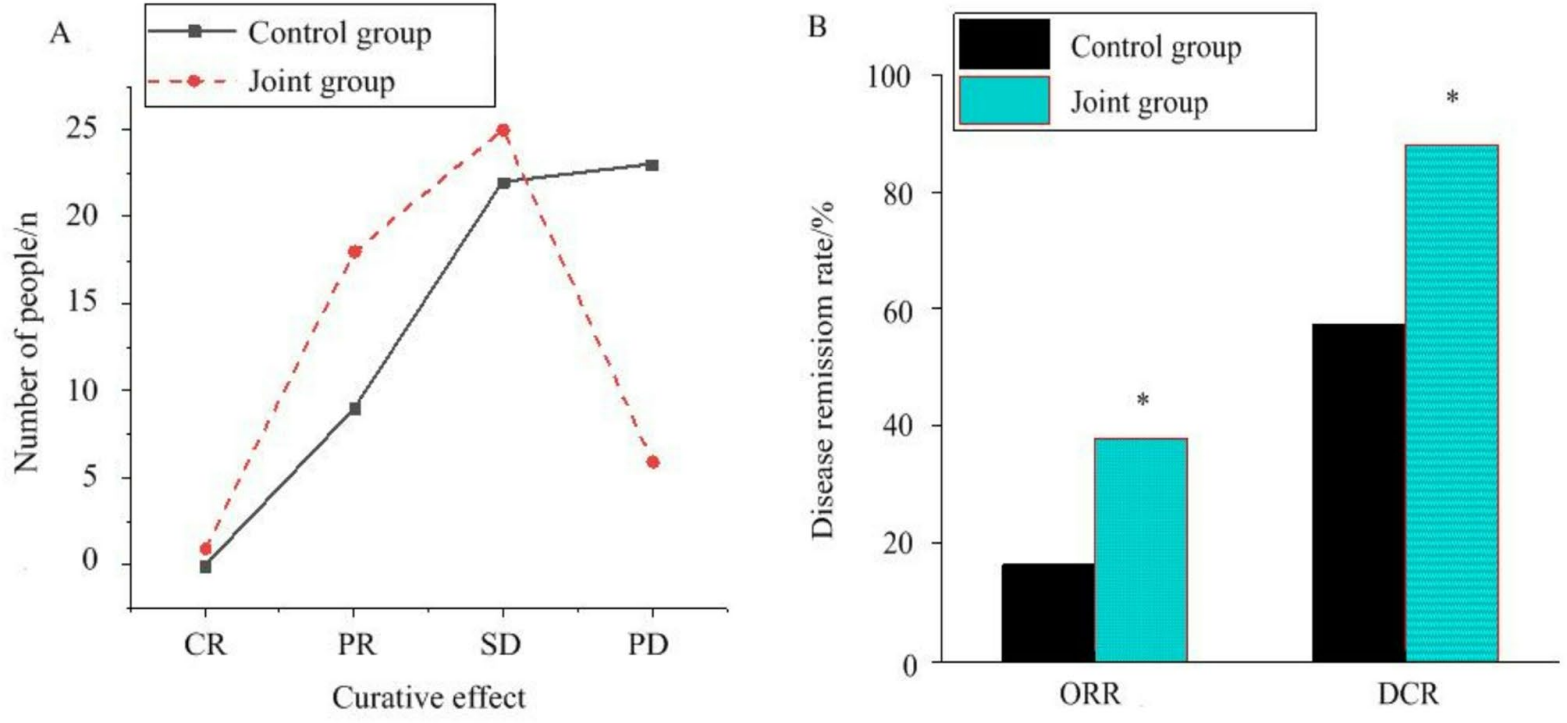
Analysis of the effect of PD-1 inhibitor plus radiotherapy on patients after treatment. (**A** is ORR; **B** is DCR). Note: *As against the CG, *P <* 0.05

The PFS was analyzed according to the iRECIST. In the CG, there were 0 individual with complete response (CR), 9 individuals with partial response (PR), 22 individuals with stable disease (SD), and 23 individuals with confirmed progressive disease (PD). In the JG, there were 1 individual with CR, 18 individuals with PR, 25 individuals with SD, and 6 individuals with confirmed PD. The ORR and disease control rate (DCR) of the JG were visibly higher as against the CG (*P <* 0.05) (Fig. 4).

### Impact of PD-1 inhibitor plus radiotherapy on the prognosis of individuals

**Fig. 5:**
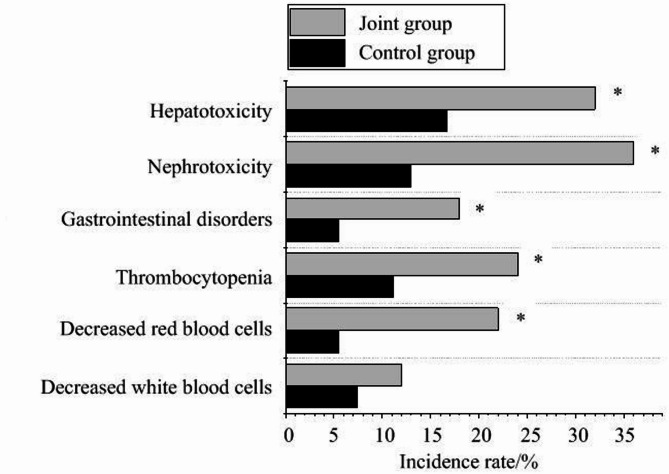
Incidence of adverse effects of PD-1 inhibitors plus radiotherapy on the prognosis of patients. Note: *As against the CG, *P <* 0.05

The adverse reactions (hepatotoxicity, nephrotoxicity, gastrointestinal dysfunction, thrombocytopenia, anemia, leukopenia) of patients after treatment were observed. The number of adverse reactions in the JG was visibly higher as against the CG (*P <* 0.05). (Fig. 5).

### Impact of EGFR expression on patient survival

**Fig. 6:**
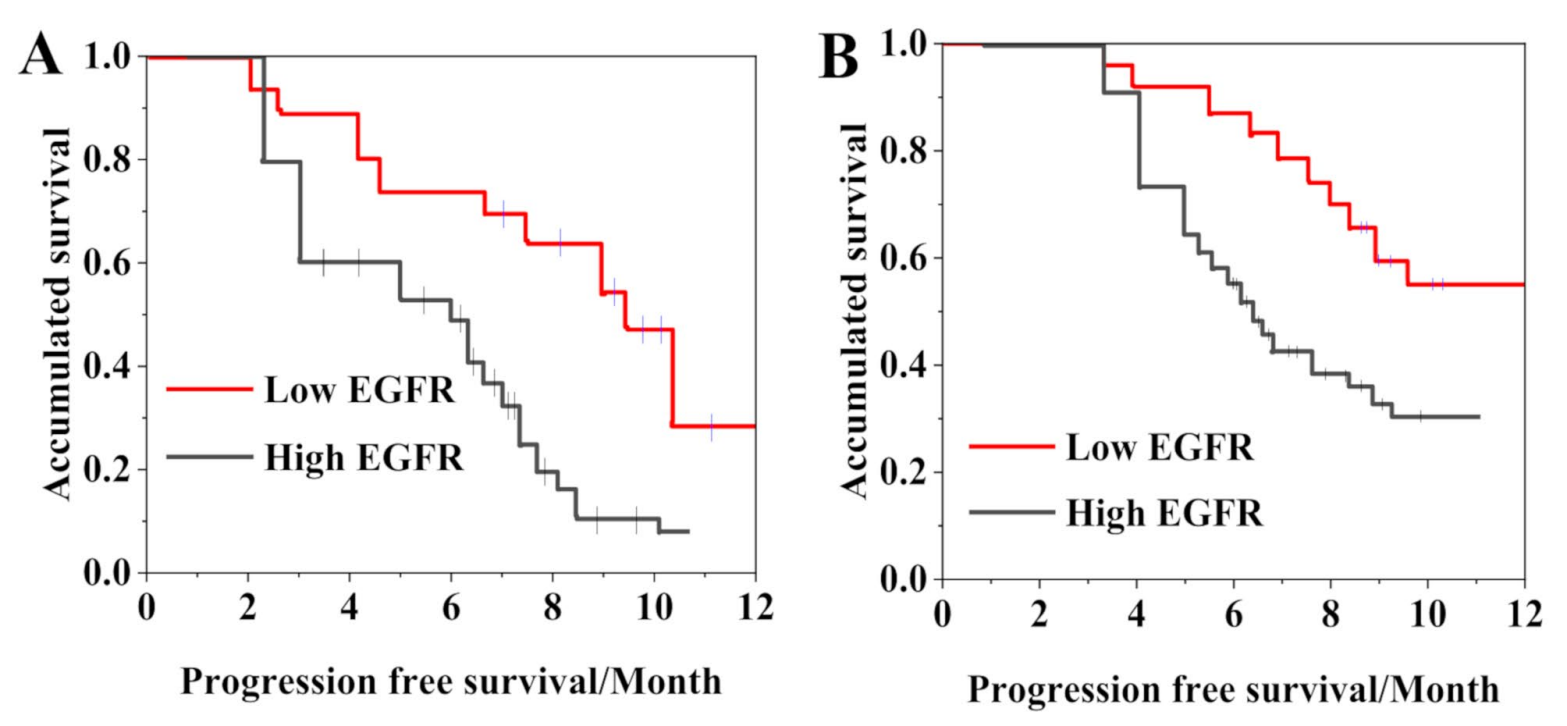
Impact of EGFR expression on patient survival. (**A** is CG; **B** is JG)

The KM method was adopted for analyzing the impact of EGFR level on survival (Fig. 6). In the CG, the median PFS was 4.8 months (2.1–5.6) in patients with low EGFR, and 3.7 months (1.0-4.2) in those with high EGFR. In the JG, the median PFS was 7.3 months (5.6–8.3) in patients with low EGFR and 5.2 months (2.9–5.9) in those with high EGFR. The median PFS of the JG was visibly longer as against the CG (*P <* 0.05).

### Multivariate analysis of PFS in BC patients

A Cox multivariate regression model was used to analyze whether EGFR expression levels serve as an independent prognostic factor prior to PD-1 inhibitor treatment in patients of the combination treatment group. The results (Table 2) show that high EGFR expression (β = 0.793, SE = 0.347) is an independent risk factor affecting patient prognosis (Wald χ^2^ = 6.384, *P* = 0.012), with a hazard ratio (HR) of 2.561 (95% CI: 1.273–4.859).

**Table 2:** Multivariate analysis of PFS in patients with BC

Factor	β	SE	Wald	*P*	HR	95%CI
EGFR	0.793	0.347	6.384	0.012	2.561	1.273–4.859

## Discussion

BC, as a major health threat to women, is undergoing a profound transformation in its treatment paradigm, shifting from traditional therapies to precision medicine [17]. This study focused on two important current developments in the field of BC treatment—immunotherapy and the synergistic effects of radiation therapy. By systematically analyzing the clinical data of 104 BC patients, the study thoroughly explored the therapeutic effects and mechanisms of PD-1 inhibitors combined with radiation therapy. The results showed that this combination treatment not only significantly improved PFS and ORR but also highlighted the differences in treatment response across various molecular subtypes of BC. Furthermore, biomarkers such as EGFR were identified as key predictors of treatment efficacy [18, 19]. The study found that the median PFS in patients receiving PD-1 inhibitors combined with radiation therapy was 8.2 months, a significant extension compared to the 5.4 months in the radiation-only group, which holds important clinical significance. In the context of solid tumor treatments, a PFS extension of more than 3 months is typically regarded as a clinically meaningful improvement. Even more encouraging, the combination treatment group achieved an ORR of 38% and a DCR of 88%, both of which were significantly superior to the control group. Notably, this therapeutic advantage was particularly pronounced in the triple-negative BC patient subgroup, which showed an ORR of 45.2%. This suggests that patients with TNBC, who have traditionally limited treatment options and poorer prognosis, may derive the greatest benefit from this combination therapy [20].

A detailed analysis of the characteristics of different molecular subtypes of BC revealed that triple-negative BC exhibited a distinctive pattern of high EGFR expression, with a positivity rate as high as 80.6%, significantly higher than the Luminal subtype (58.3%) and HER2-positive subtype (67.7%). This finding provides important insights into the differential efficacy of combination therapy. Dynamic monitoring after treatment showed that the combination therapy significantly reduced EGFR expression levels, whereas patients with baseline EGFR levels ≥ 96.21 ng/mL had a notably poorer prognosis, with a hazard ratio of 2.561. These results collectively suggest that EGFR may not only serve as a prognostic marker but also as a key biomarker for predicting the efficacy of PD-1 inhibitors combined with radiation therapy. Mechanistically, high EGFR expression may promote an immunosuppressive microenvironment through upregulation of PD-L1, while combination therapy may disrupt this immunosuppressive state, thereby improving treatment outcomes. In contrast to EGFR, the expression of P53 did not show significant changes before and after treatment; however, its baseline level was correlated with treatment response. ROC curve analysis determined the critical threshold for P53 positivity at 10%, with an AUC of 0.713, suggesting that P53 status may influence tumor sensitivity to treatment. This finding indicates that the mechanism of action of PD-1 inhibitors combined with radiation therapy may be partially independent of the p53 pathway, providing a new perspective for understanding the complex mechanisms of immunotherapy. Some studies found that PD-1 inhibitor sintilimab plus radiotherapy is very effective in the treatment of BC patients after first-line chemotherapy failure, which can greatly inhibit tumor growth. The levels of tumor markers, such as CA125, were markedly reduced, and lung metastases were also markedly reduced [21]. Some studies have shown that longer median OS and response duration are correlated with increased PD-L1 expression [22]. This article found that in the treatment of BC, PD-1 inhibitor plus radiotherapy can markedly improve the treatment effect of patients, prolong PFS, and improve the DRR and DCR. In the study of Antony et al. (2023) [23], it was found that the combination of PD-L1 inhibitors and chemotherapy could markedly reduce tumor progression by inhibiting epithelial-mesenchymal transition. Regarding safety, the study also yielded important findings. Although the incidence of adverse reactions was significantly higher in the combination treatment group, particularly for nephrotoxicity and hematologic toxicity, all adverse events were effectively controlled through standardized management strategies, such as a strict dosing interval (with a minimum of 24 h between PD-1 inhibitor administration and radiation therapy) and systematic toxicity monitoring [24]. These manageable safety characteristics provide a crucial safeguard for the clinical adoption of combination therapy.

The results of this study have multiple implications for clinical practice. First, the combination of PD-1 inhibitors and radiation therapy provides a new therapeutic option for BC patients, particularly those with triple-negative BC, who have limited treatment choices [25]. Second, pre-treatment EGFR testing may become an important tool for guiding clinical decision-making, helping to identify patient populations most likely to benefit from combination therapy [26]. Furthermore, the safety management plan established in this study provides a practical reference standard for the clinical implementation of this combination therapy. Of course, these findings also raise several scientific questions worthy of further investigation. For instance, what is the exact relationship between changes in EGFR expression levels and alterations in the immune characteristics of the tumor microenvironment? What molecular mechanisms underlie the differences in treatment response across various molecular subtypes of BC? How can treatment strategies, including radiation dosage, target area, and the timing of immune therapy, be further optimized? These are all important directions for future research [27]. Overall, this study not only confirms the clinical value of PD-1 inhibitors combined with radiation therapy in the treatment of BC, but more importantly, it reveals the predictive role of biomarkers such as EGFR, providing a new theoretical foundation and practical basis for precision medicine in BC. These findings will help drive the evolution of BC treatment toward a more personalized and precise approach, ultimately improving patient survival outcomes and quality of life. Future large-scale prospective studies are needed to validate these findings and further explore optimized combination therapy regimens, with the aim of delivering greater clinical benefits to BC patients.

## Conclusion

In conclusion, PD-1 inhibitor plus radiotherapy can markedly reduce the expression of EGFR and prolong the median PFS of patients, which has a visible effect in alleviating the clinical disease of patients. In addition, this article found that PD-1 inhibitor plus radiotherapy markedly prolonged the median PFS in patients with EGFR level ≤ 96.21ng/mL. This article provides an important reference for clinical application. However, further studies with larger samples and in-depth exploration of its mechanism of action are still needed to better guide clinical practice.

## Data Availability

All data generated or analyzed during this study are included in this published article.
